# Temporal and Spatial Properties of a Yeast Multi-Cellular Amplification System Based on Signal Molecule Diffusion

**DOI:** 10.3390/s131114511

**Published:** 2013-10-25

**Authors:** Michael Jahn, Annett Mölle, Gerhard Rödel, Kai Ostermann

**Affiliations:** 1 Institute of Genetics, Technische Universität Dresden, Helmholtzstr. 10, 01062 Dresden, Germany; E-Mails: michael.jahn@ufz.de (M.J.); annett.gross@tu-dresden.de (A.M.); gerhard.roedel@tu-dresden.de (G.R.); 2 Helmholtz Centre for Environmental Research UFZ, Department for Environmental Microbiology, Permoserstr. 15, 04318 Leipzig, Germany

**Keywords:** microbial biosensor, yeast, alpha (α)–factor, fluorescence, immobilization, agarose, modular signaling/amplification system

## Abstract

We report on the spatial and temporal signaling properties of a yeast pheromone-based cell communication and amplifier system. It utilizes the *Saccharomyces cerevisiae* mating response pathway and relies on diffusion of the pheromone α–factor as key signaling molecule between two cell types. One cell type represents the α–factor secreting sensor part and the other the reporter part emitting fluorescence upon activation. Although multi-cellular signaling systems promise higher specificity and modularity, the complex interaction of the cells makes prediction of sensor performance difficult. To test the maximum distance and response time between sensor and reporter cells, the two cell types were spatially separated in defined compartments of agarose hydrogel (5 × 5 mm) and reconnected by diffusion of the yeast pheromone. Different ratios of sensor to reporter cells were tested to evaluate the minimum amount of sensor cells required for signal transduction. Even the smallest ratio, one α–factor-secreting cell to twenty reporter cells, generated a distinct fluorescence signal. When using a 1:1 ratio, the secreted pheromone induced fluorescence in a distance of up to four millimeters after six hours. We conclude from both our experimental results and a mathematical diffusion model that in our approach: (1) the maximum dimension of separated compartments should not exceed five millimeters in gradient direction; and (2) the time-limiting step is not diffusion of the signaling molecule but production of the reporter protein.

## 1. Introduction

In the past years, advancements in the development of microbial biosensors promised technical progress to detect biologically available compounds or biohazards. A number of cell-based assays and bioreporter systems have been proposed for implementation in environmental, clinical or biotechnological monitoring [1,2]. Baker's yeast (*Saccharomyces S. cerevisiae*) is a unicellular organism suitable for whole-cell biosensor applications [3].

**Figure 1.**
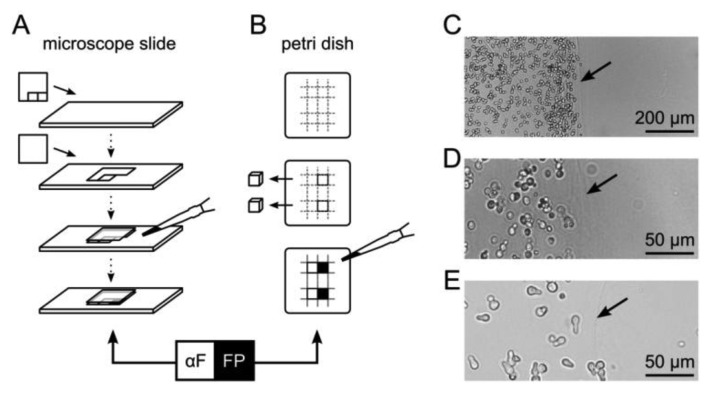
Immobilization of yeast cells in agarose compartments for microscopy and fluorescence scans. (**A**,**B**) Adjacent compartments of 5 × 5 mm were sequentially filled with agarose containing reporter cells (FP), or a source of α–factor (αF). See Supplementary Materials and Methods for details; (**C**–**E**) Microscopic imaging of the boundary region between agarose-embedded reporter cells (left) and plain agarose with diffusible α–factor (right). The boundary is visible as a blurred line (arrow). Cells were imaged immediately after immobilization (**C**,**D**) or four hours after exposure to 10 μM α–factor diffusing from the right compartment (**E**).

Many of the reported yeast sensors are based on “tailored” genetically modified yeast cells. Typically, yeast sensor cells feature an inducible or repressible promoter element that controls the expression of a reporter gene such as enhanced green fluorescent protein (EGFP), resulting in “lights on” or “lights off” signal output. Respective yeast sensor cells were reported for analytes like heavy metals [4], organic compounds [5] and hormone active substances [6,7]. All of these systems are based on a single yeast strain that senses the analyte, which drives expression of a marker gene. Recently, we reported an amplification system based on at least two different *S. cerevisiae* cell types [8]. In detail, recombinant sensor cells (cell type I) respond to the presence of an analyte by expression and secretion of α–factor. The pheromone is perceived by nearby reporter cells of mating type a (cell type II) and triggers both the natural mating response (e.g., the formation of mating projections) and concomitantly the conditional expression of a reporter gene. To this end, reporter cells carry a plasmid with the *EGFP* reporter gene under control of the α–factor-responsive *FIG1* promoter, which is upregulated approximately 100-fold within 20 min in the course of the yeast mating response [9,10].

*The most apparent change of mating S. cerevisiae* cells is the formation of mating projections resulting in a pear-like shape (“shmoo”). Yeast cells possess no structural components that render them motile. Instead of moving, cells stretch themselves towards the pheromone source (typically a mating partner) and align along the α–factor gradient [11,12]. The pheromone sensing capability of yeast is exceptionally powerful, both regarding the minimum concentration that triggers mating response and the accuracy of cell polarization. Moore *et al.* observed the formation of mating projections at concentrations as low as 10 nM (wild type) or 4 nM (hypersensitive *bar1*Δ mutants lacking the α–factor protease Bar1p) [12].

Importantly, α–factor concentration as an input signal is proportional to downstream signal output of the mating pheromone response pathway [13]. This dose-response relationship between extracellular pheromone concentration and induced gene expression is a clear advantage for a biosensor approach since input and output information can be correlated. The yeast pheromone system has been exploited in a biosensor concept, where a population of cells controls its own growth by artificial quorum sensing. Thereby, individual cells can simultaneously act as senders and receivers of the signal [14].

The recently described bimodular amplifier system [8] can be regarded as an extension of a unicellular sensor approach, in which the two functions—sensing of an analyte as the input signal and production of fluorescence as the output—are separated and connected by α–factor signaling. Particular advantages of this multi-step system are signal amplification and modularization. A single sensor cell producing the universal converter molecule α–factor can address several reporter cells, and various sensor/reporter geometries are conceivable. However, temporal and spatial properties of such a multi-modular signaling concept are unknown and may be affected by the diffusivity of the signaling molecule, the nature of the immobilization matrix, the amount of sensor cells required to activate reporter cells and reporter protein maturation.

In order to analyze this system in more detail, we examined α–factor diffusion as well as mating response and fluorescence induction in reporter cells. To this end, sources of α–factor (synthetic or cell-secreted) and pheromone-responsive reporter cells were separately immobilized in 3D compartments based on agarose hydrogel. It is easy to handle, passes optical signals and has been widely applied for cell entrapment. A concentration of 0.5% (w/v) in water is sufficient for gelation at 30 °C, and gels of 1% (w/v) are considerably rigid yet leaving average pores of 400 nm [15]. *S. cerevisiae* cells are much bigger (about 5–10 μm), while the size of α–factor peptide with a molecular weight of about 1.7 kDa is much smaller, allowing efficient entrapment of yeast cells and diffusion of pheromone molecules.

A major issue for the implementation of the bimodular system is the efficient signal transmission from pheromone-secreting cells to fluorescent reporter cells with regard to temporal and spatial performance. The diffusion and gradient formation of α–factor as the key signaling molecule is crucial and delimits the dimension of separate compartments in prospective biosensors. Here we report on the diffusion of α–factor in agarose hydrogel and its time- and space-dependent induction of spatially separated fluorescent *S. cerevisiae* reporter cells.

## 2. Experimental Section

### 2.1. Strains, Cultivation and Chemicals

*Escherichia coli* TOP10F' (Invitrogen, Darmstadt, Germany) was employed for standard cloning procedures and propagation of plasmid vectors. All yeast strains were derived from *S. cerevisiae* BY4741 *bar1*Δ [*MAT***a**, *ura3*Δ*0*, *leu2*Δ*0*, *met15*Δ*0*, *his3*Δ*1*] (EUROSCARF, Frankfurt, Germany) that naturally releases no α–factor. Transformation was performed using the protocol of Gietz and Woods [16], and recombinant cells were cultivated in selective SD medium (6.7 g/L yeast nitrogen base with ammonium sulfate, 20 g/L glucose) supplemented with 60 mg/L l-histidine, 80 mg/L l-leucine and 20 mg/L l-methionine. The pheromone α–factor was obtained from Zymo Research (Irvine, CA, USA) and low gelling point agarose from Biozym (Hessisch Oldendorf, Germany).

### 2.2. Plasmid Construction

A set of plasmids for controlled yeast pheromone signaling was described previously [8]. Briefly, constructs contain a 1.5 kb *P_ADH1_* or 1.0 kb *P_FIG1_* promoter element for constitutive and α–factor-inducible expression, respectively, of *EGFP* (fluorescence output) or *MFα1* (α–factor production). Additionally, plasmids p426ADH-TurboRFP and p426FIG1-TurboRFP were generated. The 696 bp *TurboRFP*-ORF was PCR-amplified from pTurboRFP-N (Evrogen, Moscow, Russia) using the primers 5′-TATTATACTAGTATGAGCGAGCTGATCAAGG-3′/5′-TATTATCTCGAG TCATCTGTGCCCCAGTTTG-3′ and inserted into the parental vectors p426ADH and p426FIG1 by use of the *Spe*I/*Xho*I cleavage sites (underlined). Plasmids and corresponding identifiers used in this study are summarized in Table 1.

### 2.3. Immobilization of Yeast Cells in Agarose Compartments

For microscopic fluorescence imaging, yeast cells were embedded in 5 × 5 mm compartments on a microscope slide (Figure 1A). Generic transparent adhesive tape was cut such that two squares adjacent to each other could be removed sequentially and attached to a pre-warmed glass slide (30 °C). The first square was removed and a cover slip applied. The resulting cavity was filled with 35 °C-tempered suspension consisting of 1% (w/v) agarose in SD medium with yeast cells adjusted to an optical density at 600 nm (OD_600_) of 0.75. After solidification, the second tape square was removed and the space was filled with another agarose/yeast suspension, resulting in two adjacent compartments of immobilized yeast cells. SD medium with synthetic α–factor in the second compartment served as a control.

For fluorescence scanning experiments, a Petri dish was filled to a height of five millimeters with SD medium containing 1% (w/v) agarose. Adjacent cubes of 5 × 5 × 5 mm were excised with a sterile scalpel and the resulting cavities were sequentially filled with a suspension of agarose and yeast cells (OD_600_ of 0.1, 0.5, 1.0 or 2.0), or synthetic α–factor for reference (Figure 1B).

After solidification, Petri dishes and microscope slides were incubated in a moist chamber at 30 °C for up to 48 h.

### 2.4. Fluorescence Microscopy

Microscopic slides were mounted on a Keyence BZ-8100E inverted microscope with integrated camera and automated stage (Keyence, Osaka, Japan), and 100× Plan Achromatic objective (NA1.4, WD0.13 oil immersion). Green fluorescence was detected with a GFP band pass filter (Λ_Ex_ = 472.5/30 nm, Λ_Em_ = 520/35 nm), and red fluorescence using a TxRed band pass filter (Λ_Ex_ = 562/40 nm, Λ_Em_ = 624/40 nm). A set of images was taken using eleven positions in a row with a distance of 300 μm (equals 2336 px) between each in orthogonal direction to the compartment boundary. For each position, five to ten images at different focal planes were taken and combined by *z*-projection (Keyence BZ Analyzer software).

### 2.5. Fluorescence Scanning

Square Petri dishes were analyzed with the Typhoon Trio Imager (GE Healthcare, Munich, Germany) using green laser excitation (532 nm, 20 mW), a focal plane of +3 mm and a 580BP30 band pass emission filter. Fluorescence profiles of compartments filled with pheromone-responsive reporter cells (FR) were analyzed using the “Plot Profile” tool of ImageJ [18]. A relative intensity of 1.0 corresponds to the maximum fluorescence intensity of cells displaying constitutive red fluorescence (AR) after six hours of incubation.

### 2.6. Semi-Automatic Processing of Microscopic Images

The software CellProfiler [19] was used for image processing and analysis. A pipeline of modules was created that allowed automatic identification of cells as individual objects by their size, shape and edges in bright field images. Based on the eccentricity *E* of an ellipse as the relevant shape property, cells were categorized into three shape classes: circular, elongated, or shmoo (for details see Supplementary Material). Additionally, mean fluorescence intensities of all individual cells in each image were measured.

### 2.7. Mathematical Modeling of α–Factor Diffusion

Diffusion of α–factor in a limited area was determined by a finite diffusion model [20]:

(1) $$C(x,t)=\frac{1}{2}C_{0}\sum_{n=-\infty }^{\infty }\left(\text{erf}\;\frac{h+2nl-x}{2\sqrt{D_{0}t}}+\text{erf}\;\frac{h-2nl+x}{2\sqrt{D_{0}t}}\right)$$

The diffusion coefficent *D*_0_ for α–factor was calculated based on published data by Moore *et al.* [12]. See Supplementary Material Section S3 for details. The α–factor concentration *C*(*x*, *t*) at a certain location x and time t can be determined using *C*_0_ as the initial concentration, *h* is the extent of the α–factor source (5 mm), *l* is the diffusion boundary (10 mm) and *n* is the number of iterations. The reduced diffusion in agarose hydrogels was accounted by using a correction function by Amsden [21] with values for agarose provided by Liang *et al.* [22].

## 3. Results and Discussion

### 3.1. A Two-Compartment Setup with Immobilized Yeast Cells

A bimodular biosensor like the formerly introduced multi-cellular communication and signal amplification system utilizes *functionally* separated cell types that are applied in mixtures [8]. Co-immobilization of different cell types minimizes diffusion distances of signaling molecules, such as α–factor, and may allow rapid activation of reporter cells. However, for the purpose of monitoring, such a strategy is disadvantageous due to the competition of co-localized fluorescent and non-fluorescent cells for space and nutrients. Also, regarding controlled activation of diversely functionalized cells, spatial separation can be advantageous. Thus, we tested the multi-cellular communication and signal amplification system using *spatial* separation of different cell types. Cells were entrapped in 1% (w/v) agarose hydrogel either as a thin layer on a glass slide for microscopic analysis, or in cube-shaped compartments that allowed fluorescence scanning over distances in the range of millimeters (Figure 1A,B). In both cases, two compartments containing either yeast cells, α–factor or plain agarose hydrogel were separated by a sharp boundary. This boundary was visible as a blurred line, ensuring discrimination of yeast cells belonging to the respective compartments (Figure 1C–E).

As verified by viability staining with ethidium bromide, the average cell survival rate of around 90% was not reduced by cell entrapment or during the maximum incubation time of the following experiments of six hours (Figure S1). It should be stressed that α–factor concentrations used in our system are significantly lower than those used in other studies, in which cell death was observed [23,24].

### 3.2. Diffusing α–Factor Triggers Mating Projection Formation of Reporter Cells in Agarose Compartments

**Figure 2.**
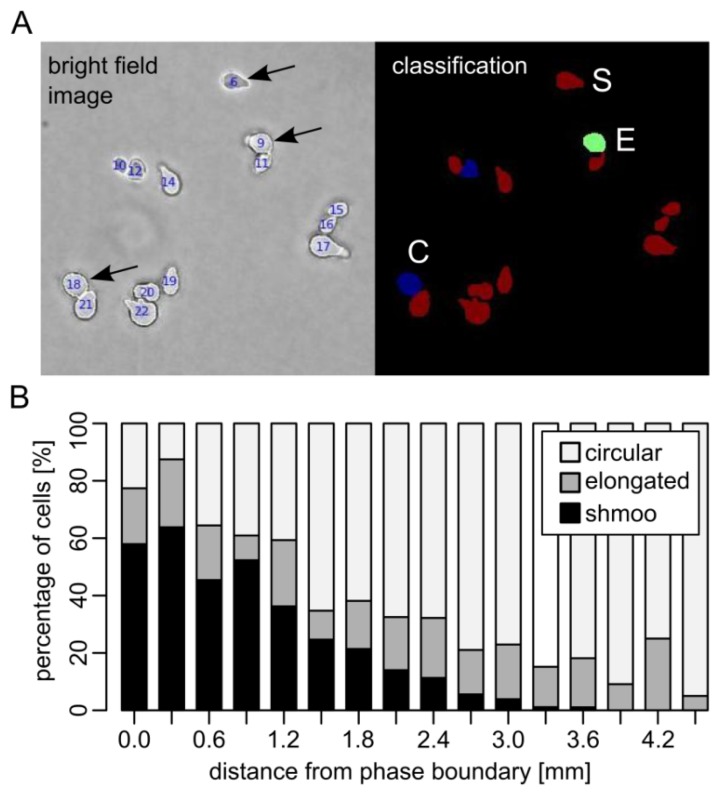
Spatial trend of mating projection formation of yeast cells exposed to diffusing α–factor. (**A**) Phenotype classification of yeast cells after α–factor treatment using ImageJ. Based on the shape property eccentricity *E*, cells were assigned to one of three classes; circular (C), elongated (E) or shmoo (S); (**B**) BY4741 *bar1*Δ cells and 10 μM α–factor were immobilized in adjacent compartments. Proportions of cell phenotypes in different distances from the compartment boundary after six hours of incubation are shown.

To examine spatial effects of reporter cell activation after immobilization, we embedded BY4741 *bar1*Δ cells and 10 μM α–factor in adjacent agarose compartments on a microscope slide and imaged cells after six hours of incubation at 16 different distances from the compartment boundary (Figure 2). The phenotype of individual cells (circular, elongated or shmoo) was evaluated by automated image analysis (Figure 2A, Supplementary Material Figure S2).

Clearly, the pheromone was able to diffuse into the adjacent compartment, thereby triggering formation of mating projections in immobilized yeast cells. We assume that α–factor progressively forms a gradient. Diffusion of α–factor was estimated using a standard diffusion model that allows to compare the theoretical pheromone concentration and its effect on cells (Supplementary Material Figure S3).

Six hours after incubation, the majority of cells close to the compartment boundary displayed definite mating projections or an elongated shape (Figure 2B). Up to a distance of three millimeters, the fraction of cells displaying a shmoo phenotype gradually decreased. Beyond this distance, mating projections could not be detected. A relatively constant proportion of elongated cells could be observed up to a distance of about four millimeters.

This graded response contrasts to recent reports about a switch-like bimodal response triggered by stable α–factor gradients [25,26]. Possibly, in contrast to the microfluidic setup used by [25,26], a dynamic α–factor gradient is formed in our two-compartment system. Since pheromone concentration and gradient slope change for every position on the slide, individual cells experience an increasing α–factor concentration over time.

### 3.3. Induction of Fluorescence in Immobilized Yeast Reporter Cells

**Figure 3.**
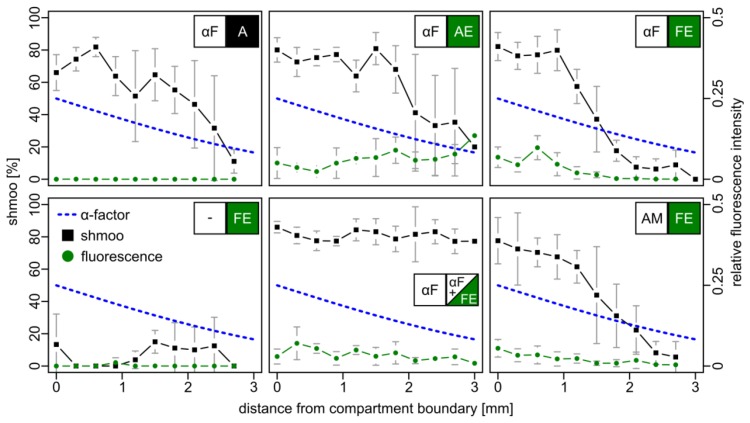
Spatial trend of shmoo and fluorescence induction in a two-compartment setup. BY4741 *bar1*Δ reporter cells (FE, see Table 1 for identifiers) and a source of α–factor (αF) were immobilized in two adjacent compartments on microscope slides and incubated for six hours prior to analysis. The percentage of shmoo-displaying cells and the average fluorescence intensity for indicated populations at different distances from the compartment containing the source of α–factor is given. The dashed line marks modeled α–factor concentration (see supplementary data). A value of 100% corresponds to the initial concentration of 10 μM. Mean values ± standard deviations from three experiments are shown.

As a further measure for pheromone detection, the induced fluorescence in BY4741 *bar1*Δ cells bearing a plasmid-borne FIG1-EGFP reporter construct (FE cells) was analyzed. Again, two adjacent compartments were mounted on a microscopic slide. One contained either cells with the empty parental vector (A cells, negative control), or cells that constitutively express *EGFP* from the moderate *ADH* promoter (AE cells, positive control), or FE reporter cells. The other compartment was filled either with 10 μM synthetic α–factor, with AE cells, or with medium without α–factor. Fluorescence and mating projections were examined after six hours (Figure 3).

With increasing distance to the compartment boundary, non-fluorescent A cells responded to the α–factor gradient with a gradually declining number of mating projections. AE cells were also characterized by such a shmoo gradient, but additionally emitted weak fluorescence irrespective of their position in the compartment. FE reporter cells exhibited fluorescence that gradually decreased with increasing distance to the boundary, accompanied by a decrease of the number of mating projections. Strikingly, a similar result was observed when the gradient was formed by α–factor secreting AM cells, indicating that the amount of released α–factor is comparable and sufficient for signaling across the two compartments with a dimension of 5 × 5 mm. In a control setup, in which synthetic α–factor was added to both compartments, uniform pheromone distribution yielded cells with uniform fluorescence and a constant fraction of the shmoo phenotype. In the absence of α–factor neither of the two effects was observed.

### 3.4. Establishing a 3D Bimodular Signaling System

Our experiments described above demonstrated that both synthetic and native α–factor is suitable and sufficient for signaling within the range of several millimeters in a reasonable response time. Both mating projection formation and EGFP fluorescence proved to be suitable indicators for the presence of α–factor, with the latter being superior in strength and reproducibility. To improve fluorescence yield and to simplify detection, the setup was modified in two ways: (1) Cubic compartments with five millimeter edge length and readout by fluorescence scanning were used instead of thin agarose layers and microscopic analysis; (2) EGFP was replaced by TurboRFP, one of the brightest fluorescent proteins presently available [24]. Preliminary results indicated that both reporters exhibit similar expression profiles in our BY4741 *bar1*Δ reporter strain (data not shown).

**Figure 4.**
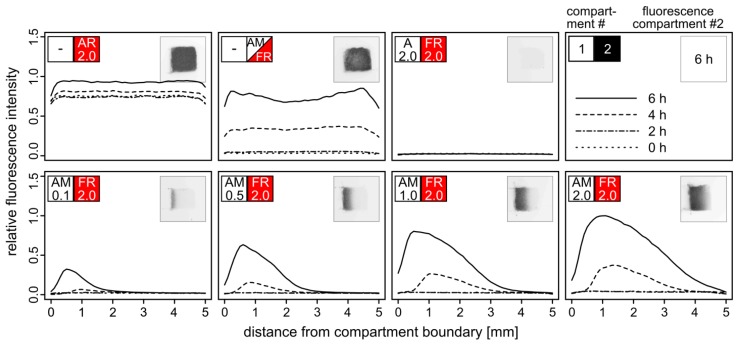
Induction of fluorescence by α–factor secreting cells in a 3D two-compartment amplifier system. Two adjacent compartments of 5 × 5 × 5 mm size in an agarose matrix were filled with plasmid-bearing BY4741 *bar1*Δ cells with an optical density OD_600_ of 0.1–2.0 (see Table 1 for identifiers). Red fluorescence of compartment 2 was detected after 0, 2, 4 and 6 h. Fluorescence scan images (small insets) and fluorescence intensity profiles (graphs) are shown. A value of 1.0 corresponds to the fluorescence intensity of a strain carrying plasmid p426ADH-TurboRFP (AR) after 6 h.

Adjacent cubic compartments were filled with yeast cells embedded in agarose hydrogel yielding different densities of α–factor secreting AM cells and TurboRFP-expressing FR reporter cells. Fluorescence of the FR compartment was monitored in intervals of two hours (Figure 4).

Yield and maximum distance from the compartment boundary of activated FR cells correlated with the density of AM cells in the adjacent compartment. Even at the lowest density of AM cells (OD_600_ = 0.1), TurboRFP expression was induced in the reporter cells at a distance of one millimeter after six hours. With higher AM cell densities, fluorescence was also observed at a greater distance from the compartment boundary, accompanied by higher and faster emerging fluorescence. AM cells at the highest tested density (OD_600_ = 2.0) triggered fluorescence in FR cells up to a distance of four millimeters. The signal peaks in a distance of about 0.5 mm from the boundary and then declines according to the diffusion profile of the pheromone. Both our observations and the diffusion model indicate that pheromone-driven gene induction does not occur beyond a distance of five millimeters within six hours. Therefore, the dimension of a single compartment within an array should not exceed five millimeters in the direction of the signal molecule gradient.

Interestingly, the maximum fluorescence observed in the two-compartment setup with the highest density of AM cells exceeded the one that was achieved by mixing AM and FR cells within the same compartment. This effect likely results from the two-fold cell density within the single compartment leading to stronger competition for resources. Irrespective of whether AM and FR were embedded separately or in a mixture, distinct fluorescence was detectable after four hours. We assume that expression of the fluorescence reporter gene and maturation of the fluorescent protein rather than α–factor diffusion *per se* is the time-limiting step [27]. Faster response times for biosensor applications should be achievable by optimizing the fluorescence reporter assay.

## 4. Conclusions/Outlook

In the course of our studies on multi-cellular sensor systems, we characterized the temporal and spatial properties of α–factor-induced mating projections and fluorescence of yeast cells. The advantages of a multi-modular signaling system are manifold. Spatial separation, in which the signal is perceived by one cell type and transmitted to another one, can increase the signal amplitude by signal amplification during the transmittance step. Furthermore, the multi-modular system provides a unique principle of specialization: the signal originating from the compartment with the responsive cell species may trigger various cellular responses of reporter or actuator cells in different adjacent, 2D- or 3D-aligned compartments, and in a concerted or sequentially triggered manner. Finally, spatial separation of signal molecule secreting sensor cells and responsive cells, e.g. fluorescence emitting reporters or enzyme producing actuators, potentially results in increased signal strength due to reduced competition for resources.

However, spatial modularization compared to direct reporter systems is at the cost of signaling speed due to the additional time required for α–factor production and secretion. With increasing distance for the signaling molecule to pass, the risk of its retention or degradation needs to be considered. Therefore, it is crucial to keep compartment dimensions as small as possible while maintaining reliable readout of the response. According to the results of our experiments, a distance of five millimeters in the direction of the gradient is both sufficient and reasonable.

In this study, the diffusion coefficient of α–factor was calculated based on earlier studies, considering the reduced diffusivity in hydrogels [12,21]. By this means, α–factor diffusion was modeled and found to be sufficiently fast on the millimeter scale. Thus, the expression and production of the reporter protein is the time-limiting step.

Our findings primarily apply for α–factor gradients in agarose, but may be easily adopted for other polymers used as immobilization matrices in biosensors. Other studies demonstrated that materials such as PEG allow diffusion of diverse model proteins [28,29]. We assume that this is also true for the considerably smaller α–factor peptide, as the ratio between a molecule's hydrodynamic radius and the matrix pore size is decisive for its diffusivity. It is also conceivable that other signaling molecules within a similar range of size can be implemented into our system. Diffusivity is higher for smaller molecules and matrices with wider pores [22], given that there is no affinity between the signal molecule and the matrix. Structural properties, e.g., pore size and chemical residues, of agarose, PEG and other hydrogels like calcium-alginate can be modified, enabling sufficient diffusion of a broad range of signaling molecules [30,31]. Therefore, our bimodular signaling and amplification system provides high flexibility and potential for a broad range of applications.

## Supplementary Material



## Figures and Tables

**Table 1.** Plasmids used in this study.

**Name (Identifier)**	**Relevant Features**	**Reference**
p426ADH (A)	1500 bp *P_ADH1_* fragment, *T_CYC1_*, *URA3* marker,	Mumberg *et al.* [17]
p426ADH-EGFP (AE)	p426ADH, *EGFP*-ORF	Gross *et al.* [8]
p426ADH-MFα1 (AM)	p426ADH, *MFα1*-ORF (YPL187W)	Gross *et al.* [8]
p426ADH-TurboRFP (AR)	p426ADH, *TurboRFP*-ORF	This study
p426FIG1-EGFP (FE)	p426ADH derivative, replacement of P*_ADH1_* with 1000bp *P_FIG1_* fragment, *EGFP*-ORF	Gross *et al.* [8]
p426FIG1-TurboRFP (FR)	p426ADH derivative, replacement of P*_ADH1_* with 1000 bp *P_FIG1_* fragment, *TurboRFP*-ORF	This study
