# Supplementary material for: Temporal and Spatial Properties of a Yeast Multi-Cellular Amplification System Based on Signal Molecule Diffusion
Source: Sensors (Basel). 2013 Oct 25;13(11):14511–22. doi: 10.3390/s131114511 (PMC3871124; doi:10.3390/s131114511)
Supplement: Supplementary file 1 [file sensors-13-14511-s001.pdf]

OPEN ACCESS

sensors

ISSN 1424-8220

www.mdpi.com/journal/sensors

Supplementary Information

Temporal and Spatial Properties of a Yeast Multi-Cellular Amplification System Based on Signal Molecule Diffusion. *Sensors* 2013, *13*, 14511-14522

Michael Jahn [1,2,†], Annett Mölle [1,†] Gerhard Rödel [1] and Kai Ostermann [1,*]

[1] Institute of Genetics, Technische Universität Dresden, Helmholtzstr. 10, 01062 Dresden, Germany; E-Mails: michael.jahn@ufz.de (M.J.); annett.gross@tu-dresden.de (A.M.); gerhard.roedel@tu-dresden.de (G.R.)

[2] Helmholtz Centre for Environmental Research UFZ, Department for Environmental Microbiology, Permoserstr. 15, 04318 Leipzig, Germany

† These authors contributed equally to this work.

* Author to whom correspondence should be addressed; E-Mail: kai.ostermann@tu-dresden.de; Tel.: +49-351-4633-6401; Fax: +49-351-4633-7725.

S1. Viability of Agarose-Embedded Yeast Cells

Viability of BY4741 *bar1*Δ cells embedded in agarose on microscope slides was determined using an ethidium bromide (EB) staining assay [1,2]. This DNA-intercalating dye is excluded by living cells but stains the nucleic acids of dead or membrane-damaged cells. EB was added during preparation of the first compartment to a final concentration of 10 µg/mL. The second compartment was filled with medium containing 1% (w/v) agarose and either 10 µM α–factor (positive control) or no α–factor (negative control). Samples were examined by fluorescence microscopy after indicated time intervals. A minimum of 50 cells per condition and time point was analyzed. Cells were counted and the proportion of stained cells—considered to be dead—was calculated.

In the absence of α–factor, constant proportions of about 10% of the cells exhibited EB-staining between zero to six hours of incubation. After 24 and 48 h, viability dropped significantly up to 35% EB-stained cells (Figure S1A), probably resulting from nutrient depletion and accumulation of waste products.

Furthermore, viability of immobilized yeast cells was determined for a distance of up to 3 mm from the compartment boundary upon exposure to 10 µM α–factor diffusing from the adjacent compartment into the cell compartment (Figure S1B). Contrary to findings in other studies that reported apoptosis of yeast cells in the presence of high α–factor concentrations [3,4], no position-dependent effect on cell

viability in the gradient was observed. The decrease in viability after 24 h and 48 h incubation was comparable to the control condition without α–factor treatment.

Figure S1. Ethidium bromide staining assay with FE reporter cells. (**A**) Dead cell fractions of control samples prior to immobilization (left) or after embedding in agarose and exposition to 10 µM α–factor (right); (**B**) Dead cell fractions of cells exposed to a dynamic α–factor gradient at 11 consecutive positions with increasing distance to the compartment boundary (0–3 mm). The dashed line marks simulated α–factor concentration (diffusion model, see text and Figure S3), where 1.0 corresponds to an initial concentration of 10 µM. Shown are mean values ± standard deviations of three independent experiments.

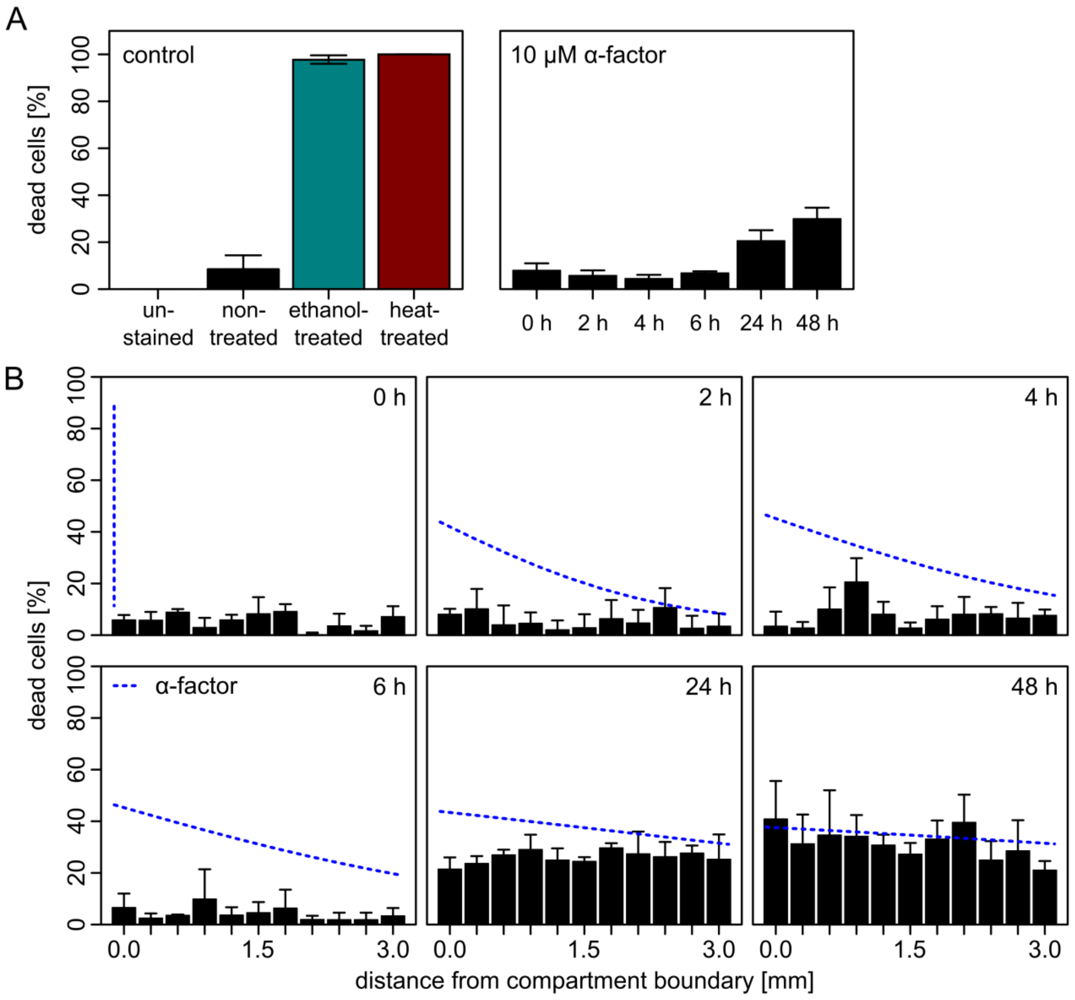

S2. Cell Classification by Object Eccentricity

Yeast cells were automatically identified and characterized using the image analysis software CellProfiler [5]. Out of ten different morphological features analyzed, the shape property "eccentricity" *E* provided the best discrimination between circular and shmoo phenotypes. *E* describes the elongation of an ellipsoid object, and is high for elongated and zero for perfectly circular objects. A set of twelve clearly shmoo-displaying cells and 12 clearly circular cells was compared to define thresholds for three cell classes: shmoo (S), elongated cells (E, intermediate state between shmoo and circular), and circular cells (C) (Figure S2).

Figure S2. Eccentricity E of yeast cells with and without mating projections. Cell morphology (n = 12 for each population) was measured with CellProfiler [5] to define the thresholds for three cell classes: shmoo ($0.67 < E \leq 1.0$), elongated ($0.57 < E \leq 0.67$), and circular ($0 \leq E \leq 0.57$).

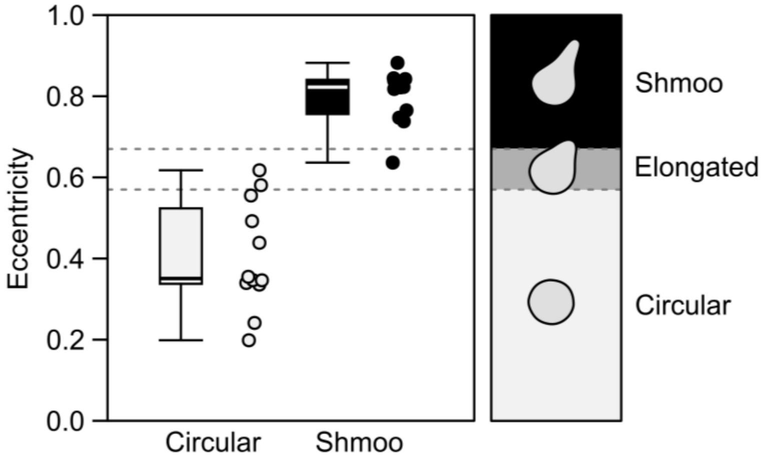

S3. Mathematical Modeling of α–Factor Diffusion

Key element in the bimodular signaling system is the tridecapeptide α–factor and particularly its diffusion properties in hydrogels. Diffusion is the process by which molecules are transported from a region of higher concentration towards a region of lower concentration by means of random molecular motion [6]. The diffusivity of small molecules like peptides and proteins is best described by the Stokes-Einstein equation:

$$D_0 = \frac{K_B T}{6\pi\eta R_H} \tag{1}$$

The diffusion coefficient D_0 states how fast a molecule or substance diffuses and has the dimension cm^2/s. K_B is the Boltzmann’s constant, T the absolute temperature (303.15 K), η is the dynamic viscosity of water as the solvent ($0.798 \cdot 10^{-3}$ Pa · s at 30 °C) and R_H is the hydrodynamic or Stokes-radius of the molecule [1]. All of these variables are known constants except the hydrodynamic radius R_H, which loosely correlates with the size of a molecule. Although many experiments employed diffusion of α–factor, its hydrodynamic radius R_H has not been determined yet. In their study, Moore *et al.* [7] examined α–factor diffusion using stable gradients and fluorescently labeled α–factor in a Y-shaped microfluidic device with a central chamber of 800 µm width. A diffusion curve provided by this study was used here to calculate the hydrodynamic radius R_H of α–factor. To this end, a finite diffusion model by Crank [6] matching the experimental setup of Moore *et al.* [7] was employed:

$$C(x,t) = \frac{1}{2} C_0 \sum_{n=-\infty}^{\infty} \left(erf \frac{h + 2nl - x}{2\sqrt{D_0 t}} + erf \frac{h - 2nl + x}{2\sqrt{D_0 t}} \right) \tag{2}$$

Here, $C(x, t)$ is the measured concentration of α–factor at a certain location (x = 135, 235, 335, 435, 535, 635 µm) and time (t = 48 s), where C_0 = 1.0 is the initial concentration, h is the extent of the α–factor source (400 µm), l is the diffusion boundary (800 µm), n is the number of iterations and D_0 is the diffusion coefficient from Equation (1). The model was fitted to the experimental data of

Moore *et al.* [7], and the hydrodynamic radius was calculated as $R_H = 1.13 \pm 0.06$, with a coefficient of determination $R^2 = 0.999$ indicating the good quality of the fit (Figure S3A).

Most likely, the diffusion coefficient of molecules in hydrogels such as agarose is reduced compared to water. Here, a mathematical model reported by Amsden [8] was employed to simulate the delayed diffusion of α–factor in agarose hydrogels:

$$\frac{D_g}{D_0} = \exp\left[-\pi\left(\frac{R_H + r_f}{k_S\varphi^{-\frac{1}{2}} + 2r_f}\right)^2\right] \tag{3}$$

This mathematical model was proven to be superior to others in terms of protein diffusion in agarose [4]. Here, D_g/D_0 is a fraction of the diffusion coefficient D_0 and indicates how strong the diffusivity of a molecule is reduced. R_H is again the hydrodynamic radius of the solute, r_f is the radius of the agarose fibers (1.9 nm), k_s is a scaling parameter (omitted) and φ is the volume fraction of agarose, calculated as mass fraction of agarose ($C_{agarose}$) divided by density ($\sigma_{agarose}$ = 1.64 g/mL) and fraction of agarose fibers ($\omega_{agarose}$ = 0.625) [4].

Figure S3. Models for diffusion of α–factor. (**A**) Fit of experimental α–factor diffusion determined by Moore *et al.* [7] (red) and a mathematical model (Equation (2), black). The grey area indicates the original α–factor distribution, the x-axis indicates the position in the microfluidics chamber used [7], and the y-axis the relative concentration of α–factor after 48 s diffusion (R^2 = 0.999); (**B**) Model of the reduced diffusivity in agarose hydrogels based on Amsden [8]. D_g/D_0 is a fraction of the diffusion coefficient D_0 for a molecule and depends on the agarose concentration ($C_{agarose}$) in the hydrogel. The diffusivity of α–factor in a 1% (w/v) agarose hydrogel is reduced to 88.7% of that in pure water (red dot); (**C**) Modeled concentration of embedded α–factor matching our experimental two-compartment setup, initial distribution in grey.

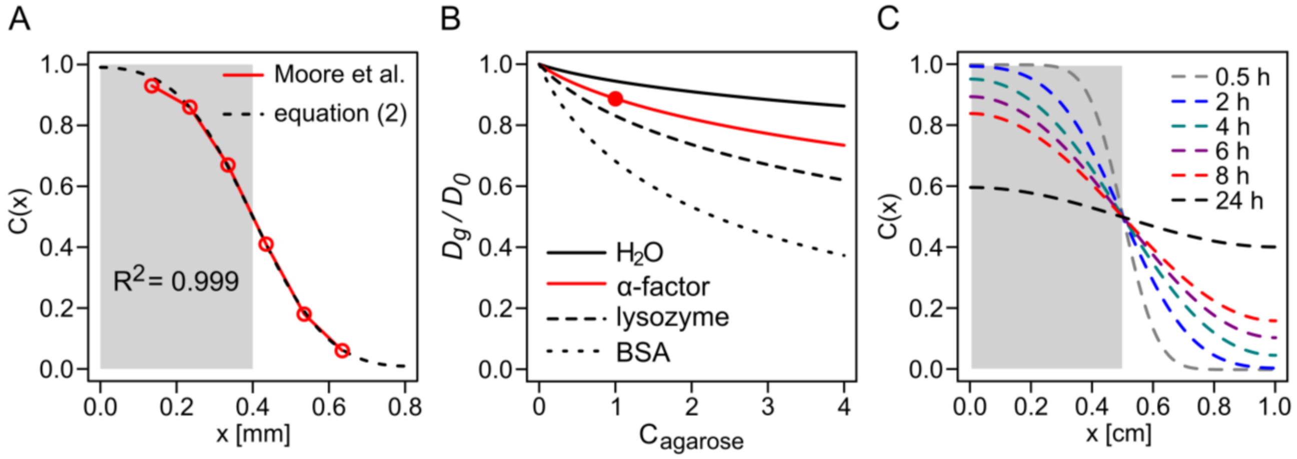

Naturally, the diffusion coefficient of molecules decreases with the increase of agarose concentration (Figure S3B). The model was applied to four different molecules: water, α–factor, and two proteins of well-known hydrodynamic radius, lysozyme (R_H = 1.9 nm) and bovine serum albumin (BSA, R_H = 3.5 nm) [4]. The impact on the diffusion coefficient is stronger for molecules with larger

hydrodynamic radii like BSA and lysozyme. The model suggests that the diffusion coefficient of α–factor in 1% (w/v) agarose is reduced to 88.7 % compared to diffusion in pure water.

Finally, the finite diffusion model (Equation (2)) and corrected diffusion coefficient of α–factor in agarose hydrogel (Equation (3)) enable to model diffusion of embedded α–factor matching our experimental two-compartment setup (Figure S3C).

Acknowledgments

This work was supported by a grant (BMBF, 03WKBH1A) of the German Federal Ministry of Education and Research to Gerhard Rödel. We thank Jennifer Gaugler for critical reading of the manuscript. We acknowledge support by the German Research Foundation and the Open Access Publication Funds of the TU Dresden.

References

1. Liang, S.; Xu, J.; Weng, L.; Dai, H.; Zhang, X.; Zhang, L. Protein diffusion in agarose hydrogel *in situ* measured by improved refractive index method. *J. Control Release* **2006**, *115*, 189–196.
2. Liegler, T.J.; Hyun, W.; Yen, T.S.; Stites, D.P. Detection and quantification of live, apoptotic, and necrotic human peripheral lymphocytes by single-laser flow cytometry. *Clin. Diagn. Lab. Immunol.* **1995**, *2*, 369–376.
3. Knorre, D.A.; Smirnova, E.A.; Severin, F.F. Natural conditions inducing programmed cell death in the yeast Saccharomyces cerevisiae. *Biochemistry (Moscow)* **2005**, *70*, 264–266.
4. Severin, F.F.; Hyman, A.A. Pheromone induces programmed cell death in *S. cerevisiae*. *Curr. Biol.* **2002**, *12*, R233–R235.
5. Carpenter, A.E.; Jones, T.R.; Lamprecht, M.R.; Clarke, C.; Kang, I.H.; Friman, O.; Guertin, D.A.; Chang, J.H.; Lindquist, R.A.; Moffat, J.; *et al.* CellProfiler: Image analysis software for identifying and quantifying cell phenotypes. *Genome Biol.* **2006**, *7*, doi:10.1186/gb-2006-7-10-r100.
6. Crank, J. *The Mathematics of Diffusion*; Oxford University Press: New York, NY, USA, 1979.
7. Moore, T.I.; Chou, C.S.; Nie, Q.; Jeon, N.L.; Yi, T.M. Robust spatial sensing of mating pheromone gradients by yeast cells. *PLoS One* **2008**, *3*, e3865.
8. Amsden, B. An obstruction-scaling model for diffusion in homogeneous hydrogels. *Macromolecules* **1999**, *32*, 874–879.

© 2013 by the authors; licensee MDPI, Basel, Switzerland. This article is an open access article distributed under the terms and conditions of the Creative Commons Attribution license (http://creativecommons.org/licenses/by/3.0/).
